# PReS-FINAL-2259: Spectrum of Sjögren syndrome in children

**DOI:** 10.1186/1546-0096-11-S2-P249

**Published:** 2013-12-05

**Authors:** BE Bica, LM Saldarriaga Rivera, H Tupinambá, MNL Azevedo

**Affiliations:** 1 Rheumatology, Universidade Federal do Rio de Janeiro, Rio de Janeiro, Brazil

## Introduction

Sjögren's syndrome is an autoimmune disease characterized by the presence of a lymphocytic infiltrate in the salivary and lacrimal glands, manifesting with xerostomia and xerophthalmia, which is more common in women between 30 and 50 years old, uncommon in children, with few cases reported in the literature.

## Objectives

To describe the clinical and laboratory presentation of pediatric patients with Sjögren's syndrome treated in primary Rheumatology Department of the Hospital Universitario Clementino Fraga Filho of the UniversidadeFederal of Rio de Janeiro, Brazil (HUCFF-UFRJ).

## Methods

26 child and adolescent patients were selected with diagnosis of primary Sjögren's syndrome, treated in the Rheumatology Department of HUCFF-UFRJ. Patients were evaluated according to modified European criteria by the American-European Consensus (VITALI et al, 2002).

## Results

26 patients were included in the study: 20 girls (76%), 6 boys (24%), with mean age at diagnosis of 12.6 years (3-21 years). Patients were 16 years of age or younger at the onset of symptoms. Eight (30.7%) patients had parotid gland enlargement as the initial manifestation of the disease, with recurrent episodes in 2 (7.6%) patients. Ten (38%) had dry mouth complaint and 16 (62%) had ocular signs and symptoms, 18 patients (69%) had altered parotid ultrasonography, 19 patients (73%) had impaired salivary glandscintigraphy and 6 patients (23%) had abnormal parotid MRI. In 8 patients (30%) the minor salivary gland biopsy showed chronic sialadenitis compatible with Sjögren syndrome. The analysis of serum autoantibodies, showed 8 patients (30%) positive for rheumatoid factor, 10 patients (38%) with Anti-Ro, 9 patients (34%) with Anti-La, 9 patients (34.6%) Anti-Ro and Anti-la and 18 patients (69%) with antinuclear antibody (ANA). 12 patients (46%) had arthritis. 7 patients (26%) had CNS involvement. 3 patients (11.5%) had vascular compromise. One patient (3.8%) showed tachyarrhythmia, leucopenia in 2 patients (7.6%), hypergammaglobulinemia in 8 patients (30%) and hypogammaglobulinemia in 1 patient (3.8%). With regard to treatment, 25 patients (96%) received hydroxychloroquine. Azathioprine was used in 4 patients (15.3%), methotrexate in 12 patients (46%), human immunoglobulin in 1 patient (3.8%), cyclophosphamide in 2 patients (7.6%), leflunomide in 1 patient (3.8%) and rituximab 2 patients (7.6%).

## Conclusion

This present study demonstrated the demographic, clinical, and therapeutic profile in a series of patients with Sjögren's syndrome in children and adolescents, a relatively rare condition, presenting an overview of this population in our hospital.

## Disclosure of interest

None declared.

